# Effect of progressive horizontal resistive force on the comfortable walking speed of individuals post-stroke

**DOI:** 10.1186/s12984-015-0007-7

**Published:** 2015-02-10

**Authors:** Christopher P Hurt, Jing Wang, Carmen E Capo-Lugo, David A Brown

**Affiliations:** Department of Physical Therapy, University of Alabama at Birmingham, 1720 2nd Avenue South, Birmingham, Alabama 35294 USA; Centre of Rehabilitation, The Second Affiliated Hospital of Jiaxing University, Jiaxing, China; Department of Neurology, Charité – Universitätsmedizin Berlin, Berlin, Germany; Department of Physical Therapy and Human Movement Sciences, Northwestern University, Evanston, USA; Interdepartmental Neuroscience Program (NUIN), Northwestern University, Chicago, Illinois USA; Department of Occupational Therapy, University of Alabama at Birmingham, Birmingham, USA

**Keywords:** Locomotion, Post-stroke, Force generation, Comfortable walking speed

## Abstract

**Background:**

Individuals post-stroke select slow comfortable walking speeds (CWS) and the major factors used to select their CWS is unknown.

**Objective:**

To determine the extent to which slow CWS post-stroke is achieved through matching a relative force output or targeting a particular walking speed.

**Methods:**

Ten neurologically nonimpaired individuals and fourteen chronic stroke survivors with hemiplegia were recruited. Participants were instructed to “walk at the speed that feels most comfortable” on a treadmill against 12 progressively increasing horizontal resistive force levels applied at the pelvis using a robotic system that allowed participant to self-select their walking speed. We compared slope coefficients of the simple linear regressions between the observed normalized force vs. normalized speed relationship in each group to a slope of -1.0 (i.e. ideal slope for a constant relative force output) and 0.0 (i.e. ideal slope for a constant relative speed). We also compared slope coefficients between groups.

**Results:**

The slope coefficients were significantly greater than -1.0 (p < 0.001 for both) and significantly less than 0 (p < 0.001 for both). However, compared with nonimpaired individuals, people post-stroke were less able to maintain their walking speed (p = 0.003).

**Conclusions:**

The results of this study provide evidence for a complex interaction between the regulation of relative force output and intention to move at a particular speed in the selection of the CWS for individuals post-stroke. This would suggest that therapeutic interventions should not only focus on task specific lower-limb strengthening exercises (e.g. walking against resistance), but should also focus on increasing the range of speeds at which people can safely walk.

## Introduction

Individuals post-stroke select very slow walking speeds [1], which result in limitations with activities of daily living [2]. These slow walking speeds may be, in part, because of lower maximum force generating capability [3,4] and caution, due to a self-perceived increased risk of falls (i.e. dynamic instability) [5]. Values of individuals’ post-stroke comfortable walking speed (CWS) range from approximately 0.2 m/s to 0.8 m/s [1,3,6,7], which are much slower than the observed walking speed of neurologically nonimpaired individuals, 1.2 to 1.5 m/s [8,9]. The CWS of nonimpaired and individuals post-stroke is highly repeatable between and within session [10,11]. The CWS, for nonimpaired individuals has been related to optimization of mechanical factors that may drive the energetic optimization of walking [12]. It is unknown however, what factors influence the walking speed of individuals post-stroke. Identification of these underlying factors can assist in developing clinical interventions that improve walking speed of these individuals.

Increasing walking speed requires greater lower limb force output [13], which results in increasing propulsive ground reaction forces [14]. Thus, the slow CWS of individuals post-stroke may be regulated with respect to their maximum force producing capacity of the lower limbs. These individuals are capable of walking at faster speeds, which suggests that they are not exerting maximum effort to walk at their CWS. The correlations between the maximal force outputs of the paretic limb and CWS [15,16] may be reflective of a preferred level of force output relative to maximum capacities [17]. Indeed, it has been suggested that these individuals scale dynamic force production with respect to a perceived maximum force output, or sense of effort [18] and that this may relate to more dynamic tasks like walking [19]. Thus, the slower walking speed may result from limitations in maximum force production, particularly by the plantarflexor and hip flexor muscle groups [20] in order to achieve a walking speed within a preferred level of effort relative to maximum force output.

Walking speed post-stroke could also be selected based on a prioritization of the nervous system. For nonimpaired individuals this prioritization may ultimately relate to energetic optimization [21], and we suggest that for individuals post-stroke, prioritization may relate to the fastest speed that confers safety and stability. The selection of an individual’s CWS could be dictated by feedforward mechanisms utilized by the nervous system to achieve a particular preferred movement pattern, resulting in the observed repeatability of an individual’s walking speed. Indeed, animal research has demonstrated the importance of the mesencephalic locomotor region in generating commands that dictate step frequency [22] that could be used as a feedforward command that sets walking speed.

We sought to design an experiment that specifically compares two hypotheses: 1) that CWS post-stroke is determined by the regulation of a relative force output associated with a particular speed, 2) that CWS post-stroke is dictated through an intention to move at a particular speed which could be related through parameters that are dictated by a particular preferred step frequency.

We describe an experiment where individuals post-stroke and age-matched nonimpaired participants walked under progressively increasing horizontal resistive forces while we observed the resulting changes in steady-state CWS. For this experiment we used a unique robotic system that allowed individuals to drive the speed of the treadmill belt based upon horizontal force production measured at the center of mass. The more fore-aft force that an individual generates in the device, the faster the treadmill belt moves, in a predictable manner. Therefore, if individuals select a walking speed relative to some targeted lower limb force output, then as external horizontal resistive force was increased, walking speed would decrease to maintain the same relative force output. However, if an individual targets a particular walking speed, then they would attempt to maintain their speed despite the increased force requirements. The two mechanisms for selecting CWS (regulation of relative force output and the intention to move a specific speed) may also coexist in some complex interaction.

Identification of the key deterministic factor, intent to move at a particular speed vs. regulation of relative force output, which results in a chosen CWS post-stroke, will help clinicians target new and innovative interventions appropriately. For example, if walking speed is selected relative to a preferred force output, then interventions that increase the range of force production will potentially increase the force generation output at a given preferred relative force output. However, if sense of speed is the determining factor, then interventions that improve energetics, safety, and/or stability at faster speeds might result in greater CWS post-stroke.

## Methods

### Participants

Ten neurologically nonimpaired individuals (age range: 41-61; 52 ± 7 years old) and fourteen chronic (>6 months post injury) stroke survivors (age range: 42-82; 54 ± 12 years old) with hemiplegia were recruited from a local database. Subject characteristics are presented in Table 1. Criteria for recruitment of nonimpaired individuals were as follows: over 40 years in age, no history of cardiac disease that would prevent them from participating in moderate exercise, no musculoskeletal, cardiovascular or neurological impairment/pathology that affected their gait performance, and the ability to walk 10 meters unassisted. Criteria for recruitment of post-stroke individuals were as follows: unilateral stroke that resulted in hemiplegia, ability to walk independently without walking aids other than ankle foot orthoses, medically stable (controlled hypertension, no arrhythmia, stable cardiovascular status), and the ability to provide written informed consent. Exclusion criteria for both groups were: history of serious cardiac disease (e.g., myocardial infarction), uncontrolled blood pressure (resting systolic pressure >140 mmHg and diastolic blood pressure >90 mmHg), presence of cerebellar and brainstem deficits, severe cognitive disorder, uncontrolled respiratory or metabolic disorders, major or acute musculoskeletal problems, and body weight greater than 250 pounds (due to robotic device weight restrictions). This study was performed at the Department of Physical Therapy and Human Movement Sciences at Northwestern University and written informed consent was obtained according to the policies of Northwestern University Institutional Review Board. Recruitment, screening, and clinical testing were completed by a research physical therapist.

**Table 1:** **Characteristics of post-stroke individuals compared with summary data from nonimpaired participants**

**Participant**	**Sex (M/F)**	**Age (years)**	**Weight (kg)**	**Height (cm)**	**Side of Paresis**	**Months Post-stroke**	**BBS max 56**	**FM max 34**	**Overground CWS**
**S1**	M	57	82	178	L	290	46	19	0.5
**S2**	F	59	66	173	R	314	53	20	0.7
**S3**	F	64	60	160	R	105	51	17	0.8
**S4**	M	57	92	180	R	105	52	26	0.9
**S5**	M	45	73	180	R	164	51	20	1.0
**S6**	M	50	83	178	L	96	49	16	0.7
**S7**	M	46	96	180	R	291	54	21	0.9
**S8**	M	42	98	173	L	27	50	15	0.8
**S9**	M	43	64	178	L	101	55	20	1.2
**S10**	M	57	95	180	R	94	53	19	0.7
**S11**	F	60	62	150	L	166	53	20	0.8
**S12**	M	53	93	178	R	46	46	21	1.0
**S13**	M	82	68	168	R	217	-	-	1.2
**S14**	M	58	77	170	L	38	47	18	1.1
**Mean**	3F/11M	55	79	173	6L/8R	147	51	19	0.9
**SD**	-	10	14	9	-	97	3	3	0.2
**Nonimpaired**	4F/6M	51	77.3	174.2	-	-	-	-	1.4
**SD**		8	14.2	9.9					0.2

SD, standard deviation; FM, Lower Extremity Fugl Meyer scores; BBS, Berg Balance Test scores; CWS, Self-selected Comfortable Walking Speed.

### Experimental procedures

After consenting for participation, all individuals were asked to complete: 1) three trials of an overground 10-meter walk test (10-MWT) at self-selected comfortable walking speed, 2) a horizontal resistive force test while walking in the robotic device the treadmill (see below), and 3) 12 walking trials at different levels of horizontal resistive forces the treadmill. Heart rate and blood pressure of the post-stroke individuals were taken before and after the data collection.

#### Experimental setup

**Figure 1:**
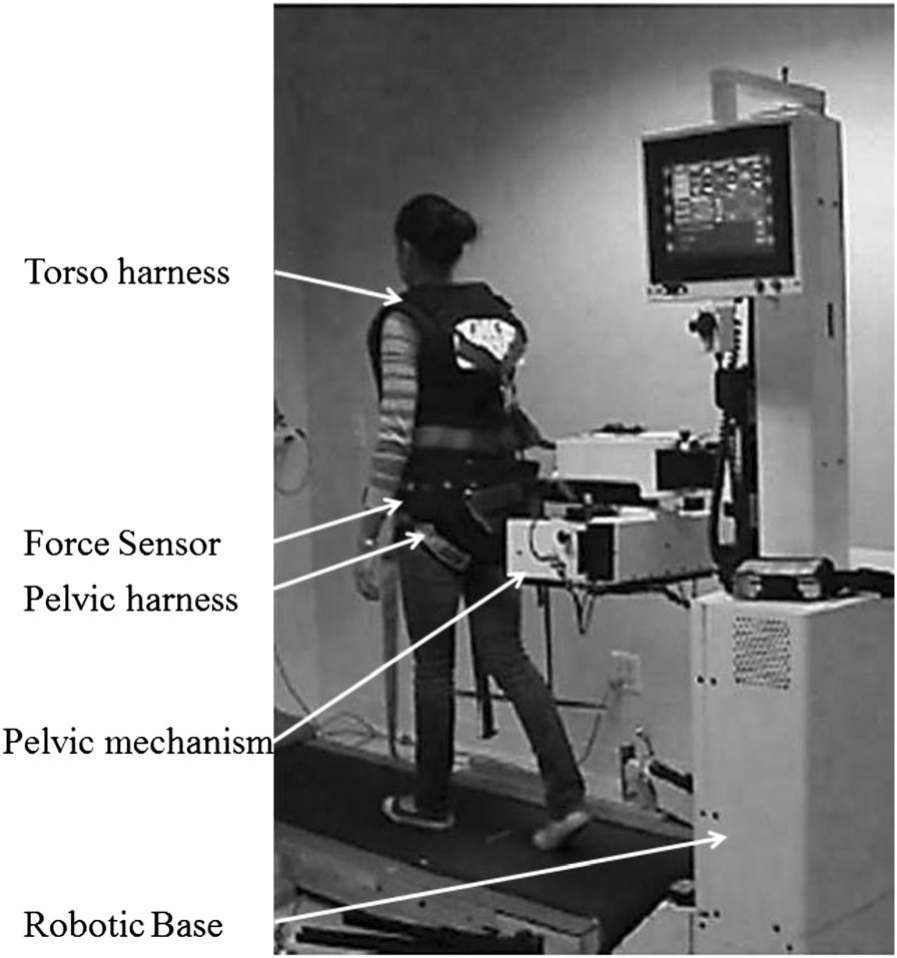
**The experimental setup used for this investigation is illustrated.** The torso harness is attached to the pelvic mechanism via a tether, which was used to limit the amount of forward trunk flexion while walking. Bilateral force sensors embedded in the pelvic interface allow the treadmill belt to be self-driven at speeds predicated by the fore-aft directed forces applied through the pelvic interface. The pelvic harness provided safety against a loss of balance while walking and did not provide any body weight support.

The experimental protocol utilized the KineAssist Gait and Balance Training System™ (KineAssist, HDT Global, Solon OH, Figure 1), which has previously been described [23,24]. Briefly, this robotic device consists of a torso and a pelvic harness attached to a mobile robotic base however, for this experiment the KineAssist was coupled with a treadmill. Bilateral force sensors embedded in the pelvic interface were integrated with a servomechanism that uses the measured horizontal force signal to dictate the treadmill belt speed based on a predictable linear relationship. Through software we created different levels of horizontal resistive force by manipulating the minimum force required to initiate motion of the treadmill belt. While this created an added force offset, the amount of additional force required for each added unit of speed was constant throughout all trials. The pelvic interface permitted movement along all degrees of freedom, which allowed the horizontal resistive forces to always be directed horizontal to the ground. Forward trunk flexion was limited to 10 degrees by a tether attached to a vest and anchored to the KineAssist in order to promote appropriate posture and to avoid undue horizontal force contributions due to body-weight. Participants underwent a short familiarization process with the robotic device in which they walked until they provided confirmation that they felt comfortable and the investigators were confident that participants could generate steady-state walking speeds.

#### Horizontal resistive force test

Participants began the horizontal resistive force test and were instructed to “walk at the speed that feels most comfortable” with zero Newtons (N) of horizontal resistive forces; each trial lasted 90 seconds and every 30 seconds the magnitude of the horizontal resistance was increased. As the horizontal resistance was increased participants were instructed “try your best to keep walking no matter how hard it gets”. A maximum of three trials were performed until a resistive force level was found that resulted in no noticeable movement of the treadmill belt. This force value was used to set the upper limit of the range of resistive force values that individuals walked against. In order to avoid fatigue, individuals were allowed to rest between trials until they confirmed that they were ready to proceed.

#### Experimental trials at randomly presented horizontal resistive force levels

Participants were instructed to “walk at the speed that feels most comfortable” against twelve randomly ordered progressively increasing horizontal resistance levels. The resistive force levels were determined relative to the highest horizontal resistance achieved in the horizontal resistance test. For instance if an individual achieved 120 N as the highest horizontal resistive force, then that individual would walk against twelve randomly presented intervals from 0-120 at 10 N increments. The order of the presented trials, from 1-12, was predetermined using an online random number generator (Random.org) and the resistive force level determined after the horizontal force test. A minimum of 20 continuous steps were collected for each trial. At least thirty seconds of rest was permitted between each trial.

### Data processing and analysis

We selected only the period of the walking trial when steady-state speed was achieved. We used custom software (MATLAB, MathWorks, Natick, MA) to determine the coefficient of variation (COV), i.e. the standard deviation divided by the mean, of a moving 10 second window of the treadmill belt speed over the duration of the trials. The 10 second period with the lowest COV was selected for processing and analysis. However, if the minimum COV exceeded 0.2 the trial was not used to quantify the horizontal resistive force vs. speed relationship because we could not assume that a steady-state speed had been achieved. After performing this analysis, the average COV between groups was 0.10 ± 0.04 vs. 0.07 ± 0.03 for post-stroke individuals and nonimpaired individuals, respectively.

The horizontal resistive forces were normalized to the highest resistive force value determined in the ‘horizontal resistance test’ (described above). Walking speed was normalized to the fastest average CWS of the 12 collected trials for a given participant. The predictable relationship between horizontal resistive force measured in the KineAssist and treadmill belt speed, coupled with the data normalization routine we performed allowed us to create a theoretical relationship where a slope of -1.0 would mean that for every unit increase in horizontal resistive force, walking speed would decrease by the same unit change. A resistive force-treadmill speed relationship such as this would suggest that individuals attempt to maintain the same relative force output while walking. The horizontal resistance vs. speed relationship was quantified by fitting a least-squares regression line to the normalized data for each subject. Only those individuals with a statistically significant linear fit were submitted to the group analysis. To test the hypothesis that CWS post-stroke is determined by a relative force output we compared the slope coefficients of each group to a slope of -1.0 with a one sample t-test. To test the hypothesis that the CWS post-stroke was dictated through an intention to move at a particular speed, we compared the slope coefficients to a slope of 0.0 with a one sample t-test. A slope of 0 would indicate that participants were maintaining their CWS regardless of the increased resistive force. To determine whether the horizontal resistive force vs. speed relationship differed between groups (nonimpaired vs. post-stroke), we utilized a multiple linear regression in which a dummy variable was coded into the model and subsequently used to create an interaction term (resistive force x group). All tests used a p < 0.05 value to determine statistical significance. All statistical tests were performed with IBM SPSS (Armonk, New York).

## Results

### Baseline walking speeds between groups

Individuals post-stroke walked at a slower speed compared to nonimpaired individuals in both the overground CWS and the observed CWS with minimal or no resistance in the KineAssist. A significant difference was observed between the overground CWS for post-stroke and nonimpaired individuals (0.88 ± 0.20 m/s vs. 1.36 ± 0.16 m/s, respectively p < 0.001). Similarly, a significant difference was also observed between the CWS at low resistance levels in the KineAssist between post-stroke and nonimpaired individuals (0.83 ± 0.18 m/s vs. 1.16 ± 0.14 m/s, respectively, p < 0.001).

### Horizontal resistance versus CWS relationship

**Figure 2:**
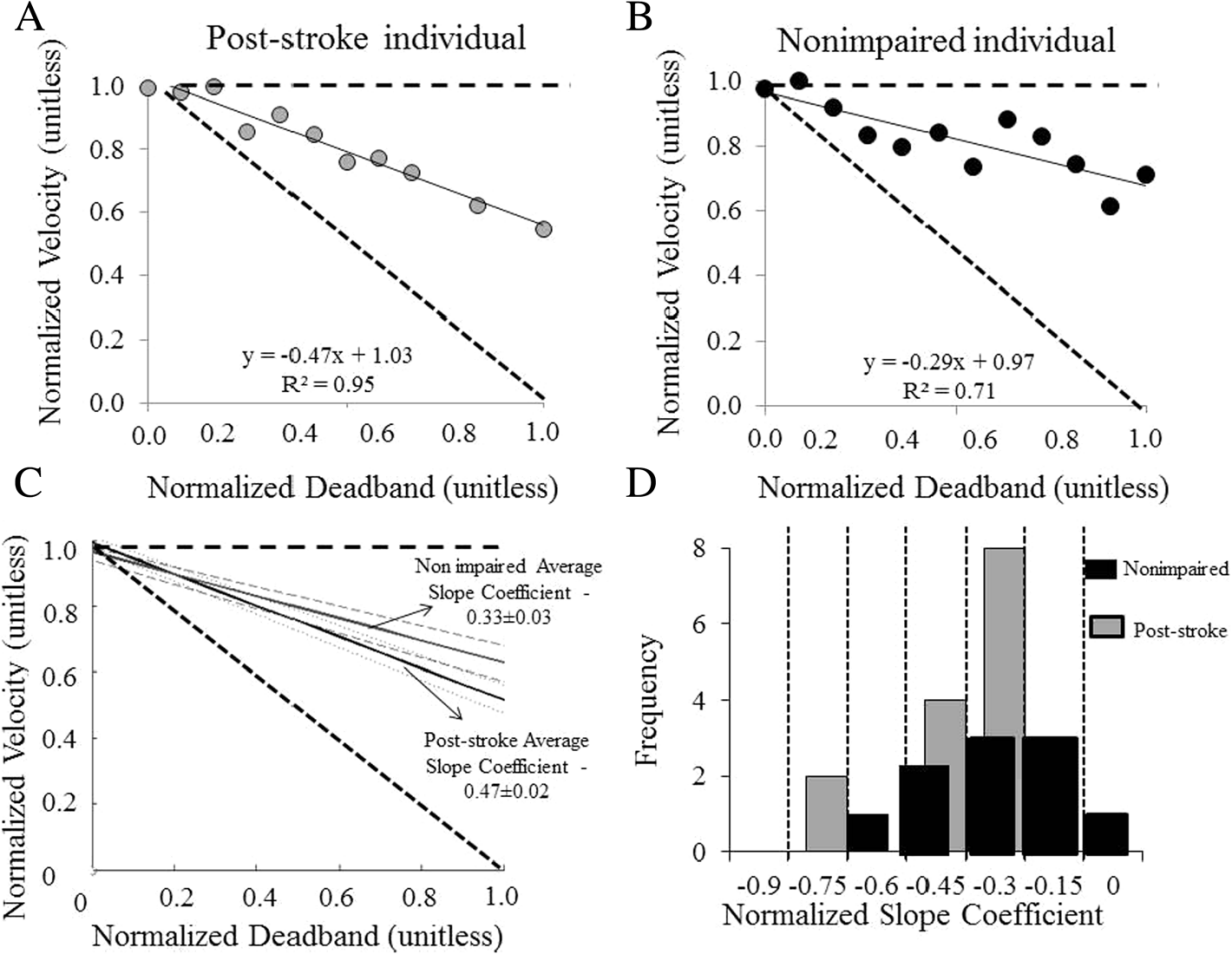
**Results of the calculated horizontal resistive force vs. CWS relationship for post-stroke and nonimpaired individuals are displayed.** Panels **A** and **B** show the linear horizontal resistive force vs. CWS relationship of an individual post-stroke and a nonimpaired individual. Panel **C** represents the average slope coefficients between the post-stroke and nonimpaired group. The difference in slopes between groups were significantly different (p = 0.02). The black dashed lines represent slope equal to zero and negative one. The thick black line represents the mean slope coefficient of individuals post-stroke. The standard deviation is represented by the grey dotted lines for nonimpaired individuals and the grey dashed line for individuals post-stroke. Panel **D)** A histogram relating slope coefficients of the horizontal resistive force-CWS relationship between individuals post-stroke (gray) and nonimpaired individuals (black).

The linearity of the relationship between horizontal resistive force and walking speed was verified for each participant (Table 2). For one nonimpaired individual, there was not a linear relationship so this participant was subsequently omitted from further analysis. Individuals neither set walking speed relative to force output nor did they clearly attempt to target a particular speed against progressively higher resistance for either group. A linear relationship was observed between the horizontal resistance force vs. walking speed for both groups (Figure 2A and B). However, slope coefficients for both groups were significantly greater than -1.0 (p < 0.001 for both) and significantly less than 0.0 (p < 0.001 for both).

**Table 2:** **Results of individual linear fits on the relationship between horizontal resistive force and comfortable walking speed**

**Post-Stroke**	**R** ^**2**^	**p value**	**NonImpaired**	**R** ^**2**^	**p value**
S1	0.68	0.002	S1	0.88	<0.001
S2	0.91	<0.001	S2	0.71	0.001
S3	0.78	<0.001	S3	0.78	<0.001
S4	0.68	0.001	S4	0.88	<0.001
S5	0.75	0.001	S5	0.00	0.933
S6	0.93	<0.001	S6	0.95	<0.001
S7	0.94	<0.001	S7	0.49	0.011
S8	0.63	0.019	S8	0.62	0.002
S9	0.82	<0.001	S9	0.50	0.014
S10	0.93	<0.001	S10	0.54	<0.010
S11	0.66	0.003			
S12	0.68	0.011			
S13	0.89	<0.001			
S14	0.96	<0.001			

Data for both groups R^2^ and whether the linear fits reached statistical significance are denote.

Both the nonimpaired and post-stroke group slowed down in response to increased horizontal resistive force. The average slope coefficient for nonimpaired individuals and individuals post-stroke was -0.37 ± 0.04, and -0.47 ± .03, respectively. This difference was statistically different between the two groups (p = 0.003, Figure 2C). Slope coefficients of both groups ranged from -0.76 to -0.34 and -0.62 to -0.28 for the post-stroke and nonimpaired group, respectively (Figure 2D).

## Discussion

In this investigation we tested the extent to which slow CWS of individuals post-stroke is selected through matching a relative force output or by targeting a preferred speed of movement. We also tested these hypotheses in individuals without impairment. If a relative force output was being utilized to select the CWS, we hypothesized that as horizontal resistive force was increased, walking speed would decrease by a specified amount to maintain the same relative force output. However, if individuals were targeting a particular preferred speed of movement, then they would attempt to maintain their walking speed even when greater forces were required. The results of this study do not support either hypotheses but instead provide evidence for a complex interaction between the utilization of a relative sense of force and an intention to move at a particular speed in the selection of the CWS for groups.

### Force effort control as a factor in determining CWS

The results of our experiment did not support the hypothesis that selection of CWS with respect to a relative force output is a primary control parameter that limits walking speed post-stroke. Investigations utilizing force matching tasks for neurologically intact individuals and individuals post-stroke have provided evidence that force output may be regulated centrally by scaling the descending motor command with respect to the perceived maximum force output, i.e. sense of effort [18,25]. In one investigation, individuals post-stroke were asked to target a force that was within the force generating capacity of both limbs, while feedback was provided about the total force output [18]. Under both the dynamic and static force matching tasks, the nonparetic limb still produced significantly more of the total force. However, when the force output was normalized to the bilateral maximum force generating capabilities, the difference in force output between limbs was not significant, suggesting that the force output of each limb was scaled relative to a sense of maximum effort. Other investigations have provided evidence that individuals scale behavior relative to their maximum force output while walking [17,19,26]. These investigations estimated the maximal dynamic force output of the hip, knee and ankle on a dynamometer and related those to respective moments recorded while walking. Compared to nonimpaired individuals, individuals post-stroke selected a slower CWS, however, these slower walking speeds equated to a similar percent of maximum effort between groups [17]. Further, despite the asymmetries in force output that were observed, individuals post-stroke walked with a similar level of bilateral effort [26].

For the present investigation, we directly tested the degree that increasing the relative force output influenced the CWS by manipulating the required effort to achieve a particular speed. For both groups, reductions in walking speed observed at higher horizontal resistance levels were much less than would be predicted if the relative force hypothesis was supported, i.e. slope coefficient greater than -1.0. Between groups, individuals post-stroke slowed to a greater degree in response to increasing resistance levels. This result may relate to the differences in maximum force generating capacity between groups. However, the present results do suggest that individuals post-stroke are capable of generating much higher horizontal force compared to their unimpeded CWS. This is important because it demonstrates that these individuals have the resources to generate greater horizontal forces to achieve faster walking speeds.

### Speed control as a factor in determining CWS

Based on our results, intention to move at a particular speed does not appear to be the primary control parameter dictating CWS. It has been suggested that CWS can result from a feedforward neural command embedded within timing circuits that influence central pattern generators [27,28]. Many investigations have provided evidence of preferred patterns of movement that are speed dependent for nonimpaired individuals [27,28]. One investigation showed that step frequency adapts quickly, less than 2 seconds, to rapid increases in treadmill belt speed [27]. Alterations to step frequency were interpreted as adjustments performed to quickly approximate a step frequency that optimized energetic cost. However, in the aforementioned investigation the speed of movement was entrained by the treadmill speed and was not freely selected. In the current investigation, no a priori information was provided about the particular resistance level that individuals would experience for each trial, thus individuals could have relied on a default preprogrammed motor command to dictate their walking speed. Individuals were instructed to “walk at the speed that felt most comfortable”, thus it is plausible that they could have relied on a generalized motor program related to their unconstrained CWS. Neither group was observed strictly adhere to a particular speed while walking against greater horizontal resistance forces, which is not surprising given the required effort to do so, particularly at the higher resistive force levels.

The greater decrease in speed in the post-stroke group could relate to the well documented decrease in force generating capacity of individuals post-stroke [15,16]. An alternative explanation is that the application of an external resistive force to the pelvis may have acted to further destabilize the post-stroke group, which are already reportedly less dynamically stable then nonimpaired controls [29] at their self-selected walking speed. A potential adaptation to this added instability would be to walk slower, which is a common adaptation to walking under potentially destabilizing conditions [30,31]. However, the relationship we report between walking speed and horizontal resistive force for all individuals post-stroke was strongly linear with slopes greater than -1.0. That means that the added unit change to horizontal resistive force resulted in walking speeds that did not decrease with the same unit change (see Figure 2A). If stability was truly a concern, one could predict that speed would decrease to a greater extent than observed to the destabilizing force (e.g. slope less than -1), ensuring that a stable gait pattern was maintained. Thus, we feel the results of this study provide evidence for a complex interaction between the regulation of relative force output and intention to move at a particular speed in the selection of the CWS for individuals post-stroke. Without the benefit of further examination, the particular reasons why individuals post-stroke select their CWS cannot be fully explained.

### Limitations of study

We recognize several limitations of this investigation. We did not assess peak isokinetic strength of our participants using a more traditional measure of peak muscular strength, an isokinetic dynamometer. We suggest that future studies could relate force output characteristics while walking with more traditional, muscle group-specific measures of peak strength. Further, we may have measured a smaller range of resistance than individuals were capable of achieving. Individuals CWS recorded at the highest resistance conditions were, on average, approximately 50% of the speed in the low to no horizontal resistance force walking trials for both groups, 46% vs.55% p = 0.10 for individuals post-stroke and nonimpaired, respectively. However, this worked to our advantage because we avoided any potential nonlinear effects that may result at more fatiguing levels of force. Individuals were tested over a relatively large range of resistance conditions though, allowing for the characterization of the horizontal resistive force vs. CWS relationship. We were also unable to measure ground reaction forces for each limb independently. Thus, for individuals post-stroke, we were unable to differentiate contributions between the paretic and nonparetic limbs to walking speeds under the progressive horizontal resistance conditions. This would be interesting because, in another experiment, as force requirements increased for a walking task, the greatest contribution of the paretic limb to the total propulsive impulse occurred at the highest effort levels [32]. In that investigation, individuals post-stroke walked overground in the KineAssist at a fixed slow speed while the relative effort, that is, the percent of maximum forward directed horizontal pushing force, was manipulated. Thus, further investigations that enable us to independently measure force output from the lower limbs is warranted. Finally, this investigation was performed while individuals walked on the treadmill. Small kinetic and kinematic differences have been detected between treadmill and overground walking between nonimpaired [33] and individuals post-stroke [34]. However, these investigations have concluded that when speed is controlled these modes of walking are similar.

## Conclusions

The results of this investigation have furthered our knowledge related to the selection of CWS of individuals post-stroke. More specifically we suggest that, although impairment of force generation is present for these individuals, this is likely not the primary factor limiting their CWS. On the contrary, the individuals that participated in our study freely chose to increase lower limb force output to walk at a speed faster than what would be predicted if they were trying to maintain a relative force output. Our results suggest that there is a complex interaction in the selection of the CWS between targeting a relative force output and the targeting of a particular speed, perhaps related to the amount of reserve capability to generate a wide range of speeds. This would suggest that therapeutic interventions may not only focus on lower-limb strengthening exercises (e.g. walking against resistance), but may also focus on increasing the range of speeds at which people can safely walk.
